# Geographic and Socioeconomic Disparity of Gastric Cancer Patients in Canada

**DOI:** 10.3390/curroncol28030190

**Published:** 2021-05-28

**Authors:** Leila Cattelan, Feras M. Ghazawi, Michelle Le, François Lagacé, Elham Rahme, Andrei Zubarev, Denis Sasseville, Ivan V. Litvinov, Kevin A. Waschke, Elena Netchiporouk

**Affiliations:** 1 Division of Dermatology, McGill University, Montreal, QC H4A 3J1, Canada; leila.cattelan@mail.mcgill.ca (L.C.); michelle.le@mail.mcgill.ca (M.L.); francois.lagace@mail.mcgill.ca (F.L.); andrei.zubarev@muhc.mcgill.ca (A.Z.); denis.sasseville@mcgill.ca (D.S.); ivan.litvinov@mcgill.ca (I.V.L.); 2 Division of Dermatology, University of Ottawa, Ottawa, ON K1N 6N5, Canada; feras.al-ghazawi@mail.mcgill.ca; 3 Division of Clinical Epidemiology, McGill University, Montreal, QC H4A 3J1, Canada; elham.rahme@mcgill.ca; 4 Division of Gastroenterology, McGill University, Montreal, QC H4A 3J1, Canada; kevin.waschke@mcgill.ca

**Keywords:** gastric cancer, gastric adenocarcinoma, geographic clustering, epidemiology, incidence in Canada

## Abstract

Gastric cancer is the 5th most common malignancy worldwide, representing ~5–10% of all new cancer cases. Although its incidence is declining, it is estimated that 1 in 98 Canadians will develop gastric cancer in their lifetime. The epidemiology and distribution of gastric cancer throughout Canada, however, remains poorly understood. A retrospective analysis of demographic data across Canada between 1992 and 2010 was performed using 2 population-based cancer registries. The incidence of gastric cancer was examined at the levels of provinces, cities, and postal codes. In addition, 43,955 patients were diagnosed with gastric cancer in Canada between 1992 and 2010; 66% were male and the average age of diagnosis was 68.4 years. The age-adjusted incidence rate was 5.07 cases per 100,000 individuals per year. The incidence decreased over the study period by 30%. High incidence rates were identified in rural areas of Newfoundland and Labrador, New Brunswick, and Quebec. Our study found a significant association between gastric cancer incidence rates and lower socioeconomic status, as well as Hispanic ethnicity. This is the first study to provide a comprehensive analysis of the incidence of gastric carcinoma in Canada, identifying high-risk populations that may benefit from increased primary and secondary prevention.

## 1. Introduction

It is estimated that 1 in ~98 Canadians will develop gastric cancer, usually adenocarcinoma, in their lifetime [1,2]. It is more common in older men [3]. Gastric cancer is the 5th most common and the 3rd most deadly malignancy worldwide and is associated with socioeconomic injustice [3,4]. Its incidence varies greatly worldwide with the highest age-standardized incidence rates (ASIR) in Asia and South America [3]. Furthermore, a strong racial disparity is seen with ASIR of 10.7 per 100,000 individuals-year in Caucasian men vs. 20.8 per 100,000 in men of Asian/Pacific origin in the US [5]. Low socioeconomic status (SES), tobacco smoking, obesity, a diet high in meat, salty and processed foods, and finally, *H. pylori* infection are strongly associated with gastric cancer development and mortality [2,3,5,6,7,8,9,10,11,12]. In particular, SES may be a predictive risk factor of gastric cancer incidence due to the underlying occupational and lifestyle exposures. A recent study of Surveillance Epidemiology and End Results (SEER) data between 2000 to 2014 in the United States found that gastric cancer incidence was ~30% higher in lowest SES neighborhoods, when contrasted with highest SES neighborhoods [13]. A study conducted in Italy in 2011 demonstrated that in nearly all regions, over 25% of deaths secondary to gastric cancer could have been avoided if the population with a low to medium education level had a mortality rate that matched that of the population with a higher education level [14].

In support of the global effort to study health disparities due to racial and socioeconomic factors [15,16], we focused on identifying the geographic distribution of gastric cancer incidence across Canadian provinces and territories and correlated incidence rates with ethnicity, SES, and other health determinants.

## 2. Materials and Methods

This study was conducted in accordance with the CISS-RDC-668035 and 13-SSH-MCG-3749-S001 protocols approved by the Social Sciences and Humanities Research Council of Canada (SSHRC) and the Quebec Inter-University Centre for Social Statistics (QICSS). An exemption from the McGill University Research Ethics Board review was obtained, in accordance with the institutional policy.

### 2.1. Data Extraction

Data were obtained from the Canadian Cancer Registry (CCR) and Le Régistre Québécois du Cancer (LRQC). International Classification of Disease (ICD) for Oncology ICD-O-3 codes were used for 10 subtypes of gastric adenocarcinoma (Table 1) similar to our prior studies [17,18,19,20,21,22,23,24,25,26,27,28,29,30,31,32,33,34]. Cases of gastric cancer were defined as the following: Adenocarcinoma (8140), intestinal adenocarcinoma (8144), diffuse adenocarcinoma (8145), tubular adenocarcinonma (8211), papillary adenocarcinoma (8260), mucinous adenocarcinoma (8480), signet ring cell carcinoma (8490), medullary carcinoma (8512), adenosquamous carcinoma (8560), and undifferentiated carcinoma (8020).

The CCR includes all cancer cases for 12 provinces and territories (excluding Quebec), diagnosed between 1992 to 2013. LRQC contains Quebec data for years 1992 to 2010. Since data from the LRQC database for Quebec were only available up to 2010, we chose to limit the analysis for this study between 1992 and 2010, for consistency. These databases provide demographic, geographic, and clinical information including sex, year, and age at diagnosis, province/city/forward sortation area (FSA). In Canada, postal codes consist of letters and numbers (e.g., H3G 1A4), where the first 3 entries (FSA) define a region in the country, with more than 1600 FSAs across Canada. Rural FSAs were defined as per the classification used by the Canada Post, in which a “0” as the second character of the FSA indicates a rural region, while a second character of “1” through “9” corresponded to an urban area.

### 2.2. Data Analysis

Incidence rates and 95% confidence intervals were calculated and reported overall, by year of diagnosis and specific geographic regions that were identified by the mapping analysis. CI were based on exact Poisson tests. Statistical significance was defined by 95% CI not overlapping with that of the national average 95% CI. The national age-standardized incidence rate for gastric cancer in Canada was calculated using the direct method with WHO 2000 to 2025 as the population standard. The age-standardized incidence rates by province, FSA, and per year were calculated using the indirect method. Incidence rates were plotted, and linear regression models were used to assess trends over time.

### 2.3. Mapping Analysis

Geographic maps of Canada, indicating the place of residence of patients recorded by the CCR, LRQC, and CVS databases, were generated using the geographic information systems software (Tableau 10.3 from Tableau Software, Seattle, WA, USA). Shape files used to create the geographic maps were obtained from Statistics Canada. Only FSAs with populations of at least 5000 individuals based on the census data were selected in order to reduce the risk of false-positive hits that could inflate the incidence rate.

### 2.4. SES/Ethnicity Analysis

Since CCR/LRQC databases do not record data concerning ethnicity nor SES, these data were retrieved from the 2001 and 2006 Canadian Census of Population. The median income was used to represent SES. Each FSA was placed into 1 of 5 quintiles based on the percentage of the population that is of Hispanic ethnicity (Q1 to Q5). Q1-Hispanic contains the FSAs with the least percentage of Hispanic individuals and Q5-Hispanic contains those with the highest percentage. Quintiles were compared with one another via incidence rate ratios (IRR) and their corresponding 95% confidence intervals. In addition to the quintile analyses, FSAs with significantly higher incidence rates than the national average were placed in one group, and those with significantly lower incidence rates in another. The average percentage of Hispanic individuals for each group was calculated and compared using the student T-test. The differences were considered significant when the *p*-value < 0.05. East Asian ethnicity and SES were analyzed using the same method. The conclusions derived from this analysis should be interpreted with caution, given that SES and ethnicity data by FSA were not directly available.

## 3. Results

**Figure 1:**
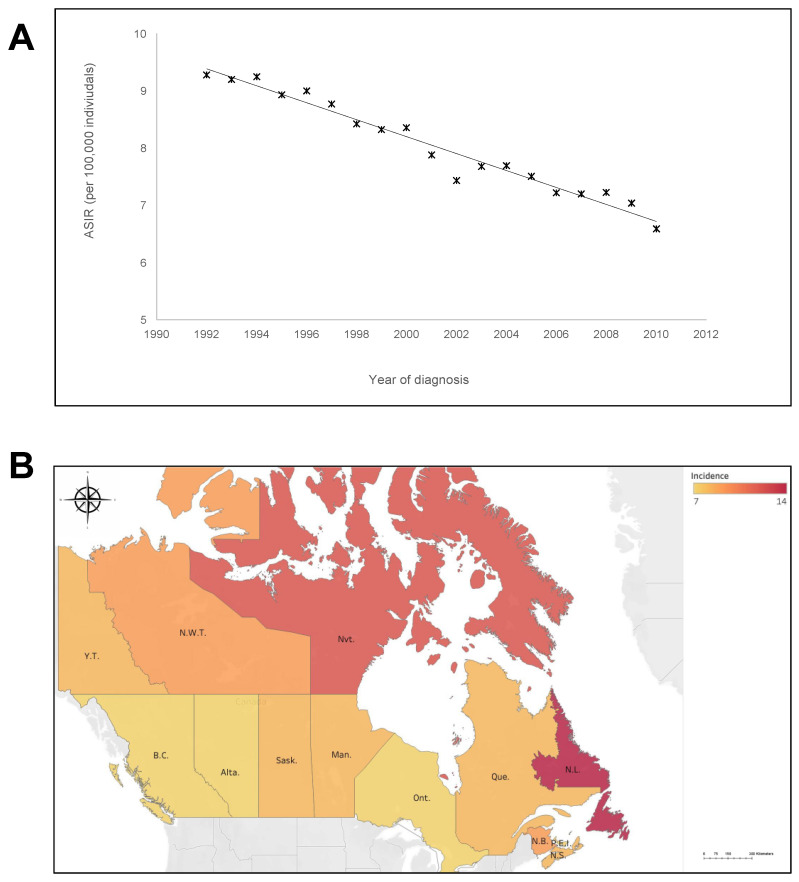
Incidence of gastric cancer throughout Canada between 1992 and 2010 over time and by province (in cases per 100,000 individuals per year) (**A**) Changing incidence rates for gastric between 1992 and 2010. (**B**) Incidence rates of gastric cancer in the Canadian provinces between 1992 to 2010.

The patients’ characteristics are presented in Table 2 total of 43,955 patients were diagnosed with gastric cancer between 1992 and 2010. The crude average national incidence rate (1992–2010) was 7.46 (95% CI: 7.39–7.53) cases per 100,000 individuals per year (Table 2) and average ASIR was 5.07 cases per 100,000 individuals per year (95% CI: 5.02–5.13). The majority of patients (66%) were males with ASIR of 6.74 (95% CI: 6.64–6.83) vs. ASIR of 3.41 (95% CI: 3.34–3.47) in females (1.95:1 male to female ratio). The average age of diagnosis was 68.4±0.5 years, with 75% of patients being ≥ 60 years. This incidence rate decreased significantly over the study period (Figure 1A) with 9.28 cases per 100,000 individual-year in 1992 and 6.59 cases per 100,000 individual-year in 2010 representing a ~30% decrease over 19 years or an annual percent change of 1.6%. The linear regression analysis incidence rate over time in [R^2^] was 0.9622; *p* = 0.002. The slope of the line is 0.148 cases per 100,000 individuals per year.

Analyses by area based-SES and ethnic composition demonstrated a significant association between gastric cancer incidence rates, SES, and Hispanic ethnicity (Table 3A,B). The incidence rates were significantly lower in the highest SES quintile compared to the lowest SES quintile (IRR_SES Q5 vs_. _Q1_ = 0.39; 95% CI 0.35–0.42 *p* < 0.001). In addition, the average median income in FSAs with statistically significant higher incidence rates was CAD 18, 700, whereas it was CAD 27,200 in FSAs with statistically significant lower gastric cancer rates (*p* < 0.001). For ethnicity, incidence rates were significantly higher in quintiles with a higher percentage of Hispanic individuals compared to quintiles with a lower percentage of Hispanic individuals (IRR_Hispanic Q5 vs_. _Q1_ = 1.47, 95% CI 1.35–1.60 *p* < 0.001). In addition, the average percentage of Hispanic individuals in FSAs with statistically significant higher incidence rates was 1.34%, whereas it was 0.62% in FSAs with statistically significant lower cancer rates (*p* < 0.001). There was no significant association between gastric cancer incidence and East Asian ethnicity (IRR_East AsiansQ5 vs_. _Q1_ = 1.02, 95% CI 0.98–1.07) nor African Canadian/black ethnicity (IRR_BlackQ5 vs_. _Q1_ = 0.93–1.13).

The national ASIR was 5.07 cases per 100,000 individuals per year (95% CI: 5.02–5.13). Geographic maps illustrate age-standardized incidence rates of gastric cancer (per 100,000 individuals per year) relative to the national average based on the CCR/LRQC databases. On a provincial level, Newfoundland and Labrador, Nunavut, New Brunswick, Manitoba, Nova Scotia, and Quebec had significantly higher ASIR compared to the national average, with the highest being in Newfoundland and Labrador at 13.71 (95% CI, 12.99–13.76) cases per 100,000 individuals per year (Figure 1B). In contrast, British Columbia and Ontario had lower ASIR of 7.00 (95% CI = 6.82–7.01), and 6.86 (95% CI = 6.75–6.86) cases per 100,000 individuals, respectively. Alberta, Saskatchewan, and Prince Edward Island’s ASIRs were on par with the Canadian average (Figure 1B).

**Figure 2:**
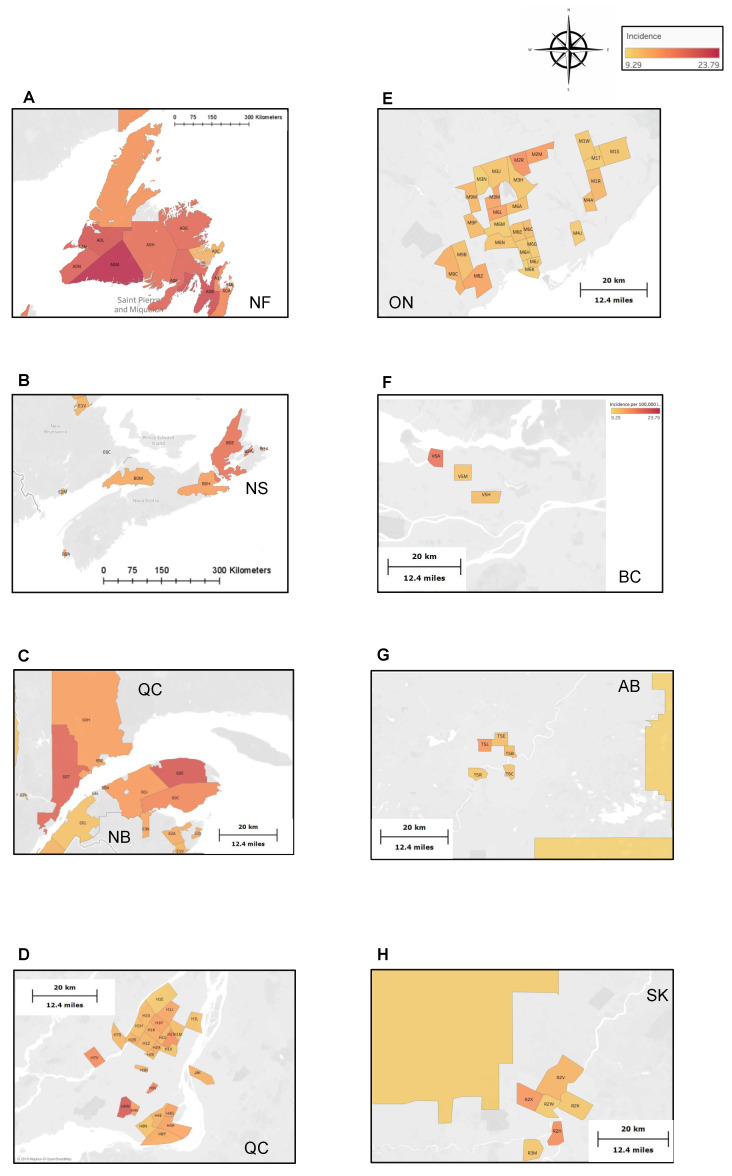
Gastric cancer high incidence FSA in the Maritimes, Quebec, Ontario, and Western Canada. Geographic maps illustrate incidence rates of gastric adenocarcinoma (cases per 100,000 individuals per year) relative to the national average based on the Canadian Cancer Registry/Quebec Cancer Registry databases. (**A**) Newfoundland and Labrador: Highest incidence FSA A0M, representing the La Poile Bay region, as well as Southwestern, Central and Northeastern rural areas of Newfoundland, Carbonea, St John’s, and finally the Avalon and Bonavista peninsula. (**B**) Nova Scotia in West Cape Breton, North Sydney, Glace Bay, Canso and Cobequid Bay. (**C**) High incidence FSAs in Eastern Quebec and New Brunswick involving Manicouagn, Le Fjord, Baie-Comeau, Rimouski, Mont-Joli, Gaspésie in Quebec, and Atholville, Bathurst, Tracadie-Sheila and Miramichi. (**D**) Montreal: Montreal-Nord, Ahunstic-Cartierville, and Villeray, Verdun, Lasalle, Côte St-Luc. (**E**) Greater Toronto Area: Scarborough, North York, and Etobicoke. (**F**) British Columbia: Vancouver. (**G**) Alberta: Edmonton. (**H**) Manitoba: Winnipeg.

Canadian cities with a population over >5000 individuals with differential (high or low) crude incidence rates are presented in Table S1. Briefly, 61/634 cities had statistically higher incidence compared to the national average. Of these, 15 (25%) were in British Columbia, 14 (23%) in Quebec, 8 (13%) in New Brunswick, 6 (10%) in Newfoundland and Labrador, and 5 (8%) in Nova Scotia. When analyzed by FSA (Table S2 and Figure 2), a similar trend was observed with many high incidence FSAs being located in Quebec (39.8%, 70/174) and Newfoundland and Labrador (9.2%, 16/174).

Eleven FSAs had an ASIRs ≥ 3-fold that of the national average (Table S3). The majority of these FSAs (6/11) were located in Newfoundland and Labrador, while the remaining ones were in rural Quebec (3/11) and Nova Scotia (2/11). The highest ASIR corresponded to the FSA A0M, representing the La Poile Bay region of Newfoundland and Labrador, with the rate of 23.79 cases per 100,000 individuals per year (95% CI, 16.99–32.39). Surrounding La Poile, additional high incidence urban and rural FSAs (Figure 2A) such as St. John’s, Carbonea, Burin, Avalon, and Bonavista peninsulas were identified. Similarly, high incidence FSAs in Nova Scotia (Figure 2B) and Quebec (Figure 2C) favored remote rural communities. When looking at major urban centers, highest ASIRs were identified in Montreal (i.e., Montreal-Nord, Ahunstic-Cartierville, Villeray, Verdun, Lasalle, and Côte St-Luc boroughs) (Figure 2D) and Toronto (i.e., Scarborough, North York, and Etobicoke) (Figure 2E). Of note, most of these FSAs correspond to racially very diverse communities as, for example, in 2006 (Canadian Census data), 45.5% of people residing in Côte St-Luc and 50% of people in Scarborough self-identified as 1st-generation immigrants [35]. Importantly, among the 11 highest incidence FSAs, 4 (36%) were home to the First Nation communities. These included the Miawpukek (A0H), We’koqma’q (B0E), Gaspé (G0E), and Essipit (G0T) communities.

## 4. Discussion

In this manuscript, we present the epidemiology of gastric cancer based on CCR and LRQS databases for all available years (1992–2010) at provincial, municipal, and FSA postal code levels. To the best of our knowledge, no previous study has assessed the distribution of gastric cancer across the entire country to identify the correlation with SES/ethnicity and identify communities where a focused intervention may be important. At the national level, we confirm a decreasing burden of gastric cancer, a male predominance, an average age at diagnosis ~68 years, and a national ASIR of 5.07 cases per 100,000 individuals per year. Similar trends are observed in other developed countries, with decreasing incidence attributed to the decline of *H. pylori* infection, decreased rates of tobacco use, and changes in food preservation [36,37]. The overall ASIR in the US between 1999 and 2013, was reported as a range from 6.6 to 7.6 per 100,000 per year [38], age of diagnosis ~70 years of age [2], and male to female incidence ratio of 2.2:1 [3].

Our geographic mapping showed significantly higher ASIRs in Newfoundland and Labrador, mostly in rural areas and often affecting First Nation communities. Various modifiable risk factors are implicated in the pathogenesis of gastric adenocarcinoma such as obesity, and diets high in salt and processed foods [10,39,40,41]. Conversely, diets plentiful in vegetables and fruit have been shown to be protective [10]. In a report by Statistics Canada in 2008, the frequency of fruit and vegetable consumption was the lowest in Newfoundland and Labrador [42]. Additionally, in 2007, this province had one of the highest rates of food insecurity in Canada (15.7%), 4th only to remote regions such as Nunavut, and Yukon [43]. In 2008, Newfoundland and Labrador demonstrated the highest rate of obesity in the country, followed by the 3 other Maritime provinces [44]. This study additionally demonstrated that obese individuals were more likely to have a low consumption of fresh fruits and vegetables, live in rural areas, and have a low income [44]. In 2015, it was found that for every 100,000 Newfoundlanders, there were 14 fast food stores and only 3 grocery stores [45]. This is in contrast to a study published in 2010 which determined that the optimal quantity of grocery stores in a trade area would be 10 per 100,000 residents [46]. In 2014, it was estimated that the cities of Montreal and Vancouver had ~13 grocery stores per 100,000 residents, Edmonton and Calgary had ~10, and Toronto had ~9 [47].

Among the 11 highest incidence FSA found in our study, 4 (36%) were home to First Nation communities. These included the Miawpukek (A0H), We’koqma’q (B0E), Gaspé (G0E), and Essipit (G0T) communities [48,49,50] supporting recent literature showing 3-fold increase in gastric cancer incidence in Northern Canadian population [51]. In 2010, it was estimated that 28% of Inuit and 27% of First Nation community members experienced moderate to severe household food insecurity vs. 8% of average Canadians [52]. In 2012, it was estimated that 42% of Inuit in Nunatsiavut residing in Newfoundland and Labrador experienced food shortages [53]. Food insecurity is linked to the adoption of an unbalanced diet, decreased physical activity, and higher prevalence of obesity [54,55,56,57]. These issues may be further exacerbated by the disproportionately high cost of fresh food in northern Canadian communities [58]. In April 2011, the Nutrition North Canada (NNC) plan was put into action, resulting in savings of approximately 5% between 2011 and 2015, however, food insecurity in these remote regions continues to persist [58]. Therefore, for these reasons, indigenous populations, as corroborated by the findings of our study, may be at higher risk of developing gastric cancer.

Our findings of higher ASIR in Canadian immigrant population, particularly in areas with significantly higher Hispanic composition (1.47, 95% CI 1.35–1.60) is aligned with the previous literature demonstrating higher rates on gastric cancer among immigrants in Ontario [59]. The Statistics Canada report showed a rapid growth of Hispanic Canadian population, by 32% between 1996 and 2001 [60]. The vast majority of Hispanic individuals reside in large urban areas such as Toronto (31%), Montreal (23%), and Vancouver (9%) [60]. These cities also displayed a high incidence of gastric cancer in the present study. Hispanic, East Asian, and African-American/black individuals, as well as other immigrant groups from countries with high rates of gastric cancer, have been shown to possess at least a 2–3 times higher prevalence of gastric cancer as compared to Caucasians in the United States [11,12,61,62]. In a study conducted between 1999 and 2013, gastric cancer incidence was mapped throughout all 50 of the United States, demonstrating that the highest gastric cancer incidence rates were found in Texas and California, both states known to have a rapid growth of Hispanic populations. Many of the identified urban high incidence FSAs correspond to the most multicultural boroughs with the highest proportion of visible minorities. By example in 2006 (Canadian Census data), ~45–50% of people residing in Côte St-Luc and Scarborough self-identified as first-generation immigrants [35], these numbers remained identical in 2016 (44.8% in Côte St-Luc and 47% in Scarborough) [63].

SES is often established from a combination of multiple variables with the 3 most prominently used being education, income, and occupation [64]. The SES analysis by examination of income was performed in our study, demonstrating a significant association between gastric cancer incidence rates and postal code areas with a low mean income. While we were unable to examine the education and occupation status in the present study, all 3 individual risk factors have been previously associated with increased incidence of gastric cancer [64]. Geographic clustering of gastric cancer cases in the northern Romagna (Italy) region between 1987 and 2008 correlated with rural, low SES communities [65]. Low SES has been previously associated with increased incidence of gastric cancer, with two thirds of cases occurring in developing nations [66], hypothesized to be due to poor sanitation and increased risk of *H. pylori* infection, as well as decreased consumption of nutritional and fresh foods due to decreased income, education, and access to resources [67,68,69].

Given that annual income does not take into account how many people are supported by that income or if one is supported by someone else’s income, it may not be the most reliable indicator to use alone [70]. When examining gastric cancer incidence by occupation, several studies have demonstrated significantly increased risk of cancer among workers in transportation, woodworking, and manual workers in comparison with those in professional work settings [64,71,72]. When assessing instead education as the independent indicator, 2 large European-based studies demonstrated that patients with lower education levels had a higher incidence of gastric cancer, with the study by Nagel et al. having overall lower smoking rates, lower body mass index, and increased vegetable intake, strengthening this observation [73,74]. In fact, as per the study by Sarkar et al., among the 3 variables defining SES, the education level appears to be the most informative and reliable predictor of gastric cancer risk [64].

Our study limitations include first and foremost the retrospective design and data years available (1992–2010). Data for the Canadian territories were sparse and not statistically significant. Due to confidentiality regulations, the data could not be presented in its entirety due to the mandatory rounding of frequencies of cases to the nearest 5 or 10, as well as the suppression of data exhibiting frequencies that were lower than 5. For the same reason, localities with less than 5000 inhabitants had to be excluded. For smaller cities/FSAs due to a relatively low number of residents and/or low number of gastric cancer cases, ASIR could not be calculated, and crude incidence rates were used. Detailed spaciotemporal information is not available through cancer registries and, hence, FSAs at inception were used. Given that the databases used did not include data concerning patient ethnicity, SES, and other health related risk factors, indirect comparison was used.

The reported associations that were found when using area-based SES and ethnic composition analysis may not be applicable when examining individual-level factors such as SES and ethnicity, as these were indirectly correlated and could not be causally linked. Finally, given that the databases employed did not provide individual data concerning nutrition habits, income, and immigrant or First Nation composition of the populations with gastric cancer in each FSA, the interpretations discussed should be taken with caution in the context of ecological fallacy, as inferences about the nature and risk factors of gastric cancer cannot be made definitively based solely upon the aggregated data. More research is needed in order to define the geographical risk factors associated with gastric cancer with certainty.

This is the first study to describe the strikingly unequal geographic distribution of gastric cancer in Canada. Importantly, localities with highest ASIR of gastric cancer are characterized by statistically higher than national average rates of known risk factors, notably low SES, and non-Caucasian ethnicity (notably Hispanic and First-Nation). The highest incidence rates in our country corresponded to rural/remote communities where high rates of food insecurity, obesity, and/or other risk factors (e.g., visible minority, low SES) are well characterized. High incidence rates in First Nation communities were confirmed [51]. Identified high-risk communities should not be ignored as it is estimated that up to 30% of gastric cancer cases may be preventable through risk factor modification [75,76]. In Japan and South Korea, where nation-wide screening programs requiring all individuals >40 to undergo annual upper endoscopic exams have had a positive impact on the incidence and mortality of gastric cancer [77,78,79]. While it may not be feasible to adopt such a widespread screening guideline for all Canadians >40, it may be beneficial to consider earlier screening in high-risk communities who present with many of the aforementioned risk factors similar to the UK guidelines [76].

## 5. Conclusions

While the incidence rates of gastric cancer are decreasing in Canada, incidence rates vary greatly on the geographic location favoring rural areas in Newfoundland and Labrador, New Brunswick, and Quebec. First-nation and highly multicultural (particularly Hispanic) communities were identified among high incidence regions. The association with low SES was confirmed. A total of 11 FSAs had an ASIR of gastric cancer >3-fold that of the national average. Since ~30% of gastric cancer cases are thought to be preventable, targeting high risk communities is of particular interest to improve disease burden and survival.

## Figures and Tables

**Table 1:** Prevalence of different subtypes of gastric adenocarcinoma analyzed in Canada between 1992 and 2010.

Gastric Adenocarcinoma Subtypes	ICD-O-3 Code	No. of Patients	Percentage of Total
Adenocarcinoma	8140	29,135	66.3
Intestinal adenocarcinoma	8144	4525	10.3
Diffuse adenocarcinoma	8145	1535	3.6
Tubular adenocarcinoma	8211	430	1.0
Papillary adenocarcinoma	8260	220	0.5
Mucinous adenocarcinoma	8480	825	1.9
Signet ring cell carcinoma	8490	6955	15.8
Medullary carcinoma	8512	5	0.01
Adenosquamous carcinoma	8560	165	0.3
Undifferentiated carcinoma	8020	160	0.3
Overall Gastric Adenocarcinoma	-	43,955	100

**Table 2:** Epidemiologic characteristics of gastric adenocarcinoma in Canada between 1992 and 2010.

Patient Demographics	Incidence
Total number of patients	43,955
By sex	
Number of males (%)	28,830 (66%)
Number of females (%)	15,125 (34%)
By age group	
<10	0 (0%)
10–19	5 (0.01%)
20–29	175 (4.0%)
30–39	990 (2.3%)
40–49	3155 (7.2%)
50–59	6490 (15%)
60–69	11,005 (25%)
70–79	13,800 (31%)
80–89	7490 (17%)
90+	825 (1.9%)
Average age	68.36 ± 0.51
Average ASIR in Males in number of cases per 100,000 individuals per year (95% CI)	6.74 (6.64–6.83)
Average ASIR in Females in number of cases per 100,000 individuals per year (95% CI)	3.41 (3.34–3.47)

**Table 3:** Quintile analysis of gastric adenocarcinoma incidence by socioeconomic status and ethnicity. Incidence rate ratios represent the ratio between the incidence rate of the given quintile and Q1.

**A: Socioeconomic Status**			
**Quintile**	**Median Income ($)**	**Incidence Rate (per 100,000)**	**Incidence Rate Ratio (95% CI)**
Q1	<20,000	9.17	-
Q2	20,000–25,000	7.35	0.80 (0.78–0.82)
Q3	25,000–30,000	5.86	0.64 (0.62–0.66)
Q4	30,000–35,000	4.43	0.48 (0.46–0.51)
Q5	>35,000	3.53	0.39 (0.35–0.42)
**B: Ethnicity**			
**Quintile**	**Percentage of Hispanic Individuals** **(%)**	**Incidence Rate** **(per 100,000)**	**Incidence Rate Ratio (95% CI)**
Q1	0.00	7.69	-
Q2	0.01–1.99	7.17	0.93 (0.89–0.98)
Q3	2.00–3.99	7.52	0.98 (0.92–1.03)
Q4	4.00–5.99	9.61	1.25 (1.15–1.35)
Q5	>6.00	11.32	1.47 (1.35–1.60)

## Data Availability

3rd Party Data. Restrictions apply to the availability of these data. Data was obtained from the Canadian Cancer Registry (CCR) and Le Registre Québécois du Cancer (LRQC) and are available from the authors with the permission of Statistics Canada.

## Supplementary Materials

The following are available online at https://www.mdpi.com/article/10.3390/curroncol28030190/s1. Table S1: Incidence of gastric adenocarcinoma in Canadian cities. Cities are divided into high incidence (**A**) and low incidence (**B**) compared to the average gastric cancer incidence rate in Canada; Table S2: List of populous forward sortation areas (FSA) in Canada with high (**A**) and zero (**B**) incidence of gastric adenocarcinoma from 1992 to 2010; Table S3: List of populous forward sortation areas (FSA) in Canada with high incidence of gastric adenocarcinoma from 1992 to 2010, sorted by their association with either urban or rural areas of the country.
