# Supplementary material for: Geographic and Socioeconomic Disparity of Gastric Cancer Patients in Canada
Source: Curr Oncol. 2021 May 28;28(3):2052–64. doi: 10.3390/curroncol28030190 (PMC8161777; doi:10.3390/curroncol28030190)
Supplement: Supplementary file 1 [file curroncol-28-00190-s001.zip › curroncol-1196539-supplementary.pdf]

Current Oncology

MDPI

Article

Geographic and Socioeconomic Disparity of Gastric Cancer Patients in Canada

Leila Cattelan [1], Feras M. Ghazawi [2], Michelle Le [1], François Lagacé [1], Elham Rahme [3], Andrei Zubarev [1], Denis Sasseville [1], Ivan V. Litvinov [1], Kevin A. Waschke [4] and Elena Netchiporouk [1,*]

[1] Division of Dermatology, McGill University, Montreal, QC H4A 3J1, Canada; leila.cattelan@mail.mcgill.ca (L.C.); michelle.le@mail.mcgill.ca (M.L.); francois.lagace@mail.mcgill.ca (F.L.); andrei.zubarev@muhc.mcgill.ca (A.Z.); denis.sasseville@mcgill.ca (D.S.); ivan.litvinov@mcgill.ca (I.V.L.)
[2] Division of Dermatology, University of Ottawa, Ottawa, ON K1N 6N5, Canada; feras.al-ghazawi@mail.mcgill.ca
[3] Division of Clinical Epidemiology, McGill University, Montreal, QC H4A 3J1, Canada; elham.rahme@mcgill.ca
[4] Division of Gastroenterology, McGill University, Montreal, QC H4A 3J1, Canada; kevin.waschke@mcgill.ca
* Correspondence: elena.netchiporouk@mcgill.ca

Citation: Cattelan, L.; Ghazawi, F.M.; Le, M.; Lagacé, F.; Rahme, E.; Zubarev, A.; Sasseville, D.; Litvinov, I.V.; Waschke, K.A..; Netchiporouk, E. Geographic and Socioeconomic Disparity of Gastric Cancer Patients in Canada. *Curr. Oncol.* **2021**, *28*, 2052–2064. https://doi.org/10.3390/curroncol28030190

Received: date
Accepted: date
Published: date

Publisher's Note: MDPI stays neutral with regard to jurisdictional claims in published maps and institutional affiliations.

Copyright: © 2021 by the authors. Submitted for possible open access publication under the terms and conditions of the Creative Commons Attribution (CC BY) license (http://creativecommons.org/licenses/by/4.0/).

Copyright: © 2021 by the authors. Licensee MDPI, Basel, Switzerland. This article is an open access article distributed under the terms and conditions of the Creative Commons Attribution (CC BY) license (http://creativecommons.org/licenses/by/4.0/).

Table S1. Incidence of gastric adenocarcinoma in Canadian cities. Cities are divided into high incidence (**A**), and low incidence (**B**) compared to the average gastric cancer incidence rate in Canada. All case numbers are rounded to the nearest 5. All population numbers are rounded to the nearest thousand.

A: Highest frequency cities.						
**City**	**Province**	**Frequency**	**Population**	**Incidence per 100,000 individuals per year**	**Lower CI (95%)**	**Upper CI (95%)**
Yarmouth	NS	50	10400	25.30	18.78	33.36
Dundas	ON	30	6240	25.30	17.07	36.12
Okanagan-Similkameen D	BC	25	5660	23.25	15.04	34.32
Inverness, Subd. A	NS	25	5940	22.15	14.33	32.70
Alnwick	ON	25	6380	20.62	13.34	30.45
Comox-Strathcona B	BC	30	8010	19.71	13.30	28.14
Cumberland, Subd. C	NS	20	5370	19.60	11.97	30.28
Côte-Saint-Luc	QC	115	31860	19.00	15.68	22.80
Bathurst	NB	45	12930	18.32	13.36	24.51
Lac-Mégantic	QC	20	5920	17.78	10.86	27.46
Trail	BC	25	7590	17.34	11.22	25.59
Gaspé	QC	50	15360	17.13	12.72	22.59
Joliette	QC	60	18510	17.06	13.02	21.96
Campbellton	NB	25	7740	17.00	11.00	25.10
Peace River	AB	20	6460	16.29	9.95	25.17
Ponoka	AB	20	6460	16.29	9.95	25.17
Dauphin	MB	25	8130	16.18	10.47	23.89
Powell River	BC	40	13060	16.12	11.51	21.95
Iroquois Falls	ON	15	5060	15.60	8.73	25.74
Asbestos	QC	20	6820	15.43	9.42	23.84
Shediac	NB	15	5280	14.95	8.36	24.66
Stephenville	NF	20	7050	14.93	9.12	23.06
Saint-Calixte	QC	15	5300	14.90	8.33	24.57
Shippagan	NB	15	5480	14.41	8.06	23.76
Vegreville	AB	15	5490	14.38	8.04	23.72
Cowichan Valley B	BC	20	7340	14.34	8.76	22.15
St. Andrews	NB	30	11020	14.33	9.67	20.45
Sidney	BC	30	11030	14.32	9.66	20.44
Inverness, Subd. B	NS	15	5600	14.10	7.88	23.25
Athabasca County	AB	20	7530	13.98	8.54	21.59
Beaver County	AB	15	5670	13.92	7.79	22.97
Donnacona	QC	15	5770	13.68	7.65	22.57
Qualicum Beach	BC	20	7710	13.65	8.34	21.09
La Sarre	QC	20	7780	13.53	8.26	20.90
Sainte-Julienne	QC	20	7820	13.46	8.22	20.79
Happy Valley-Goose Bay	NF	20	7940	13.26	8.09	20.48
Gander	NF	25	10260	12.82	8.30	18.93
Port Colborne	ON	45	18480	12.82	9.35	17.15
Dolbeau-Mistassini	QC	35	14760	12.48	8.69	17.36
Parksville	BC	25	10690	12.31	7.96	18.17
Conception Bay South	NF	50	21460	12.26	9.10	16.17
Vanier	ON	25	11110	11.84	7.66	17.48
Comox	BC	25	11120	11.83	7.66	17.47

Penticton	BC	70	31690	11.63	9.06	14.69
Moncton	NB	140	63390	11.62	9.78	13.72
Corner Brook	NF	45	20490	11.56	8.43	15.47
St. John's	NF	220	101980	11.35	9.90	12.96
Cape Breton	NS	220	105090	11.02	9.61	12.57
Edmundston	NB	35	16980	10.85	7.56	15.09
North Cowichan	BC	55	26950	10.74	8.09	13.98
Magog	QC	50	24620	10.69	7.93	14.09
Saint John	NB	140	70070	10.52	8.85	12.41
Sault Ste. Marie	ON	150	76180	10.36	8.77	12.16
Sept-Îles	QC	50	25600	10.28	7.63	13.55
Thetford Mines	QC	50	25710	10.24	7.60	13.49
Nanaimo	BC	145	76410	9.99	8.43	11.75
North Vancouver	BC	155	82430	9.90	8.40	11.58
LaSalle	QC	135	73010	9.73	8.16	11.52
Victoria	BC	140	76430	9.64	8.11	11.38
Kelowna	BC	185	102520	9.50	8.18	10.97
Niagara Falls	ON	140	80230	9.18	7.73	10.84

B: Lowest frequency cities.

City	Province	Frequency	Population	Incidence per 100,000 individuals per year	Lower CI (95%)	Upper CI (95%)
Saint-Bruno-de-Montarville	QC	70	72630	5.07	3.95	6.41
Cochrane	AB	70	73000	5.05	3.93	6.38
Mirabel	QC	85	89940	4.97	3.97	6.15
Spruce Grove	AB	25	26750	4.92	3.18	7.26
West Lincoln	ON	70	75380	4.89	3.81	6.18
Orangeville	ON	50	54060	4.87	3.61	6.42
Coaldale	AB	110	119660	4.84	3.98	5.83
Wainfleet	ON	70	76480	4.82	3.76	6.09
St. Marys	ON	50	54800	4.80	3.56	6.33
Lévis	QC	70	77790	4.74	3.69	5.98
South Glengarry	ON	20	22350	4.71	2.88	7.27
Hull	ON	115	128820	4.70	3.88	5.64
Cypress County	AB	70	79120	4.66	3.63	5.88
Kawartha Lakes	ON	45	50980	4.65	3.39	6.22
Scarborough	ON	75	85670	4.61	3.62	5.78
Bayham	ON	105	120000	4.61	3.77	5.58
Essex	ON	35	40070	4.60	3.20	6.39
Kingsclear	NB	85	98600	4.54	3.62	5.61
Ottawa	ON	20	23360	4.51	2.75	6.96
Lachenaie	QC	505	590000	4.50	4.12	4.92
Québec	QC	330	387850	4.48	4.01	4.99
L'Île-Bizard	QC	135	159670	4.45	3.73	5.27
Carignan	QC	90	107130	4.42	3.56	5.43
Saint-Hippolyte	QC	75	90190	4.38	3.44	5.49
EAST YORK	ON	15	18040	4.38	2.45	7.22
Clearview	ON	50	61780	4.26	3.16	5.62
Edwardsburgh/Cardinal	ON	15	18610	4.24	2.37	7.00
Etobicoke	ON	20	25180	4.18	2.55	6.46

Drummond/North Elmsley	ON	55	69300	4.18	3.15	5.44
Banff	AB	20	25250	4.17	2.55	6.44
Val-Bélair	QC	25	31630	4.16	2.69	6.14
Sainte-Anne-des-Monts--Tourelle	QC	15	18990	4.16	2.33	6.86
La Pêche	QC	20	25410	4.14	2.53	6.40
Olds	AB	105	134390	4.11	3.36	4.98
Barrie	ON	50	64290	4.09	3.04	5.40
Bluewater	ON	55	71220	4.06	3.06	5.29
Morinville	AB	430	559000	4.05	3.67	4.45
Ajax	ON	15	19790	3.99	2.23	6.58
Saint-Élie-d'Orford	QC	635	848000	3.94	3.64	4.26
Saint-Basile-le-Grand	QC	15	20100	3.93	2.20	6.48
East Zorra-Tavistock	ON	375	503880	3.92	3.53	4.33
Delson	QC	10	13450	3.91	1.87	7.20
Cantley	QC	80	108000	3.90	3.09	4.85
Aurora	ON	10	13510	3.90	1.87	7.16
St. Clair	ON	270	365140	3.89	3.44	4.38
Central Okanagan	BC	15	20750	3.80	2.13	6.28
Middlesex Centre	ON	80	111760	3.77	2.99	4.69
Stone Mills	ON	60	84490	3.74	2.85	4.81
Fort Saskatchewan	AB	10	14120	3.73	1.78	6.86
Candiac	QC	30	43960	3.59	2.42	5.13
Loyalist	ON	10	14730	3.57	1.71	6.57
Greater Napanee	ON	10	14790	3.56	1.70	6.54
South-West Oxford	ON	10	14830	3.55	1.70	6.53
Trois-Rivières-Ouest	QC	10	14880	3.54	1.69	6.51
Okotoks	AB	10	15080	3.49	1.67	6.42
Wheatland County	AB	10	15110	3.48	1.67	6.41
Kenora	ON	10	15260	3.45	1.65	6.34
Port Hope and Hope	ON	15	23090	3.42	1.91	5.64
Dollard-des-Ormeaux	QC	10	15460	3.40	1.63	6.26
Vaudreuil-Dorion	QC	10	15680	3.36	1.61	6.17
Bois-des-Filion	QC	10	15910	3.31	1.58	6.08
Fleurimont	QC	30	48020	3.29	2.22	4.69
Minto	ON	15	24370	3.24	1.81	5.34
Brant	ON	10	16390	3.21	1.54	5.91
Centre Wellington	ON	20	32880	3.20	1.95	4.94
Blainville	QC	15	24700	3.20	1.79	5.27
Fort Frances	ON	25	41410	3.18	2.06	4.69
Springwater	ON	10	16640	3.16	1.51	5.82
Terrebonne	QC	60	100510	3.14	2.40	4.04
Fort St. John	BC	10	16770	3.14	1.50	5.77
Quinte West	ON	25	42220	3.12	2.02	4.60
Grande Prairie	QC	25	42620	3.09	2.00	4.56
Cavan-Millbrook-North Monaghan	ON	5	8530	3.09	0.99	7.20
Le Gardeur	QC	10	17260	3.05	1.46	5.61
Whitecourt	AB	5	8670	3.04	0.98	7.08
Gatineau	QC	145	253740	3.01	2.54	3.54
Val-des-Monts	QC	5	8760	3.00	0.97	7.01

Saint-Augustin-de-Desmaures	QC	10	17710	2.97	1.42	5.47
Strathmore	AB	5	8880	2.96	0.96	6.92
Masson-Angers	QC	5	8890	2.96	0.95	6.91
Airdrie	AB	15	26950	2.93	1.64	4.83
Stoney Creek	ON	30	54000	2.92	1.97	4.17
Foothills County	AB	10	18020	2.92	1.40	5.37
Taché	MB	5	9050	2.91	0.94	6.79
Saint-Colomban	QC	5	9080	2.90	0.93	6.76
Sainte-Julie	QC	15	27450	2.88	1.61	4.74
Grey Highlands	ON	5	9200	2.86	0.92	6.68
Carleton Place	ON	5	9210	2.86	0.92	6.67
Leeds and the Thousand Islands	ON	5	9240	2.85	0.92	6.65
Lorraine	QC	5	9360	2.81	0.91	6.56
Oro-Medonte	ON	10	18780	2.80	1.34	5.15
Prévost	QC	5	9470	2.78	0.90	6.48
Dartmouth	NS	35	67570	2.73	1.90	3.79
Hinton	AB	5	9690	2.72	0.88	6.34
Saint-Luc	QC	10	19470	2.70	1.29	4.97
Woolwich	ON	10	19580	2.69	1.29	4.94
Brighton	ON	5	9910	2.66	0.86	6.20
Rideau Lakes	ON	5	9950	2.64	0.85	6.17
Varennes	QC	10	20110	2.62	1.25	4.81
Milton	ON	25	50460	2.61	1.69	3.85
Lakeshore	ON	15	30670	2.57	1.44	4.25
Whitehorse	YT	10	20490	2.57	1.23	4.72
Scugog	ON	10	20500	2.57	1.23	4.72
Clarence-Rockland	ON	10	20560	2.56	1.23	4.71
Yellowknife	NT	70	144220	2.55	1.99	3.23
Saguenay	QC	10	20730	2.54	1.22	4.67
Amherstburg	ON	5	10420	2.53	0.81	5.89
Temiskaming Shores	ON	5	10750	2.45	0.79	5.71
South Dundas	ON	5	10850	2.43	0.78	5.66
The Nation Municipality	ON	5	10910	2.41	0.78	5.63
Erin	ON	5	10990	2.39	0.77	5.59
Williams Lake	BC	5	11210	2.35	0.76	5.48
Wellington North	ON	5	11320	2.32	0.75	5.43
Kincardine	ON	5	11450	2.30	0.74	5.36
Guelph/Eramosa	ON	5	11950	2.20	0.71	5.14
West Grey	ON	5	11960	2.20	0.71	5.13
Saugeen Shores	ON	20	48440	2.17	1.33	3.36
Wood Buffalo	AB	20	49280	2.14	1.30	3.30
Cap-Rouge	QC	5	16120	1.63	0.53	3.81
Saint-Nicolas	QC	5	17120	1.54	0.50	3.59
South Frontenac	ON	5	17940	1.47	0.47	3.42

Table S2. List of Populous Forward Sortation Areas (FSA) in Canada with high (**A**) and zero (**B**) incidence of gastric adenocarcinoma from 1992 to 2010. All population numbers are rounded to the nearest ten.

A: High Incidence						
**FSA**	**Province**	**Frequency**	**Population**	**Age- Standardized Incidence Rate per 100,000 Individuals per Year**	**Lower 95% CI**	**Upper 95% CI**
A0M	NF	40	8850	23.79	16.99	32.39
A0B	NF	95	23610	21.18	17.13	25.89
A0L	NF	30	7670	20.59	13.89	29.39
H4W	QC	90	23300	20.33	16.35	24.99
G0E	QC	45	11790	20.09	14.65	26.88
A0N	NF	55	14860	19.48	14.67	25.36
A0E	NF	105	29000	19.06	15.59	23.07
G0T	QC	60	16790	18.81	14.35	24.21
A0G	NF	140	39840	18.50	15.56	21.83
A0H	NF	70	20470	18.00	14.03	22.74
V6A	BC	55	16530	17.51	13.19	22.79
B0E	NS	100	30100	17.49	14.23	21.27
B2A	NS	30	9070	17.41	11.74	24.85
J1T	QC	25	7860	16.74	10.83	24.71
H3V	QC	20	6550	16.07	9.81	24.82
G6B	QC	25	8280	15.89	10.28	23.46
V1R	BC	30	9950	15.87	10.70	22.65
A0A	NF	160	53640	15.70	13.36	18.33
G0C	QC	155	52350	15.58	13.23	18.24
H7V	QC	60	20490	15.41	11.76	19.84
B1S	NS	25	8550	15.39	9.96	22.72
R2H	MB	45	15480	15.30	11.16	20.47
H1T	QC	85	29600	15.11	12.07	18.69
A1B	NF	50	17450	15.08	11.19	19.88
M2R	ON (Toronto)	110	38430	15.06	12.38	18.16
T9C	AB	20	7010	15.02	9.17	23.19
B1A	NS	50	17680	14.88	11.05	19.62
A1Y	NF	15	5320	14.84	8.30	24.48
L8R	ON (central)	30	10650	14.83	10.00	21.17
A0K	NF	100	35830	14.69	11.95	17.87
G5H	QC	20	7260	14.50	8.85	22.39
R2X	MB	45	16360	14.48	10.56	19.37
T5L	AB	45	16390	14.45	10.54	19.34
A2N	NF	25	9110	14.44	9.34	21.32
A1E	NF	80	29400	14.32	11.36	17.82
E2L	NB	30	11050	14.29	9.64	20.40
G6G	QC	55	20420	14.18	10.68	18.45
M2M	ON (Toronto)	80	29990	14.04	11.13	17.47
H4V	QC	55	20620	14.04	10.57	18.27
G8L	QC	35	13190	13.97	9.73	19.42
G0Y	QC	25	9450	13.92	9.01	20.56
G0J	QC	95	36050	13.87	11.22	16.96
M6L	ON (Toronto)	55	20950	13.82	10.41	17.99
H4G	QC	75	28700	13.75	10.82	17.24
B5A	NS	35	13400	13.75	9.57	19.12
G0H	QC	30	11610	13.60	9.17	19.42
H1P	QC	45	17420	13.60	9.92	18.19
B0H	NS	30	11630	13.58	9.16	19.38
V0S	BC	25	9710	13.55	8.77	20.00

H4H	QC	50	19480	13.51	10.03	17.81
G7X	QC	70	27310	13.49	10.52	17.04
M8Z	ON (Toronto)	35	13670	13.48	9.38	18.74
P0A	ON (Northern)	40	15710	13.40	9.57	18.25
M3M	ON (Toronto)	60	23730	13.31	10.15	17.13
H1J	QC	25	9900	13.29	8.60	19.62
V8A	BC	45	17930	13.21	9.63	17.68
V0X	BC	50	19960	13.18	9.78	17.38
V1Y	BC	80	31970	13.17	10.44	16.39
R7N	MB	25	10090	13.04	8.44	19.25
J9Z	QC	20	8090	13.01	7.94	20.10
E3N	NB	35	14170	13.00	9.05	18.08
J9E	QC	20	8100	13.00	7.93	20.07
L8K	ON (central)	80	32510	12.95	10.27	16.12
H8P	QC	55	22410	12.92	9.73	16.81
A1C	NF	35	14280	12.90	8.98	17.94
A1V	NF	25	10260	12.82	8.30	18.93
H2A	QC	45	18510	12.80	9.33	17.12
B0M	NS	45	18570	12.75	9.30	17.07
H1R	QC	70	29060	12.68	9.88	16.02
V8S	BC	40	16630	12.66	9.04	17.24
S4P	SK	30	12480	12.65	8.53	18.06
M9C	ON (Toronto)	85	35630	12.56	10.03	15.53
G1E	QC	60	25180	12.54	9.57	16.14
E1X	NB	30	12620	12.51	8.44	17.86
E2A	NB	45	18940	12.50	9.12	16.73
G5C	QC	30	12740	12.39	8.36	17.69
J4K	QC	60	25480	12.39	9.46	15.95
L8T	ON (central)	45	19270	12.29	8.96	16.45
H2B	QC	40	17150	12.28	8.77	16.72
P7B	ON (Northern)	70	30130	12.23	9.53	15.45
H3N	QC	70	30160	12.22	9.52	15.43
J0V	QC	60	25860	12.21	9.32	15.72
L2T	ON (central)	25	10780	12.21	7.90	18.02
H4E	QC	70	30230	12.19	9.50	15.40
J8H	QC	30	13050	12.10	8.16	17.27
T2G	AB	25	10900	12.07	7.81	17.82
R2V	MB	70	30570	12.05	9.39	15.23
H1Z	QC	80	34950	12.05	9.55	14.99
S0A	SK	130	56930	12.02	10.04	14.27
S4H	SK	25	10980	11.98	7.75	17.69
A0C	NF	35	15380	11.98	8.34	16.66
G0R	QC	190	83590	11.96	10.32	13.79
G6V	QC	80	35200	11.96	9.48	14.89
E1V	NB	30	13220	11.94	8.06	17.05
M1R	ON (Toronto)	65	28730	11.91	9.19	15.18
P3E	ON (Northern)	60	26580	11.88	9.07	15.29
J3R	QC	30	13310	11.86	8.00	16.94
G0B	QC	30	13320	11.85	8.00	16.92
M9M	ON (Toronto)	45	19980	11.85	8.65	15.86
G5L	QC	70	31210	11.80	9.20	14.91
H1G	QC	110	49110	11.79	9.69	14.21
H1S	QC	50	22340	11.78	8.74	15.53
H7G	QC	45	20140	11.76	8.58	15.74

H2E	QC	55	24670	11.73	8.84	15.27
E1C	NB	65	29160	11.73	9.05	14.95
J6E	QC	85	38200	11.71	9.35	14.48
M6C	ON (Toronto)	55	24730	11.71	8.82	15.24
G0M	QC	80	36030	11.69	9.27	14.54
V9K	BC	30	13530	11.67	7.87	16.66
M9P	ON (Toronto)	45	20350	11.64	8.49	15.57
T5B	AB	35	15890	11.59	8.07	16.12
G1V	QC	40	18170	11.59	8.28	15.78
M4A	ON (Toronto)	30	13670	11.55	7.79	16.49
J6S	QC	50	22830	11.53	8.55	15.20
G1K	QC	40	18270	11.52	8.23	15.69
G1L	QC	50	22920	11.48	8.52	15.14
H1M	QC	60	27510	11.48	8.76	14.78
H1H	QC	75	34700	11.38	8.95	14.26
M6A	ON (Toronto)	40	18530	11.36	8.12	15.47
V0H	BC	110	50980	11.36	9.33	13.69
G7B	QC	40	18620	11.31	8.08	15.40
G7H	QC	65	30260	11.31	8.72	14.41
H1L	QC	70	32650	11.28	8.80	14.26
L3K	ON (central)	40	18660	11.28	8.06	15.36
V2A	BC	75	35040	11.27	8.86	14.12
M6E	ON (Toronto)	85	39790	11.24	8.98	13.90
V5H	BC	70	32850	11.22	8.74	14.17
T5E	AB	65	30550	11.20	8.64	14.27
M9B	ON (Toronto)	60	28220	11.19	8.54	14.40
P6B	ON (Northern)	50	23620	11.14	8.27	14.69
V8V	BC	50	23770	11.07	8.22	14.60
T1A	AB	55	26160	11.07	8.34	14.40
L2E	ON (central)	45	21410	11.06	8.07	14.80
E2M	NB	40	19130	11.01	7.86	14.99
H1X	QC	60	28820	10.96	8.36	14.10
G0N	QC	45	21630	10.95	7.99	14.65
J1X	QC	55	26440	10.95	8.25	14.25
T6C	AB	40	19230	10.95	7.82	14.91
J1G	QC	40	19250	10.94	7.81	14.89
H8N	QC	55	26490	10.93	8.23	14.22
J0G	QC	40	19270	10.93	7.80	14.88
S0G	SK	130	62640	10.92	9.13	12.97
M6N	ON (Toronto)	85	41110	10.88	8.69	13.46
V5M	BC	45	21790	10.87	7.93	14.54
G0L	QC	130	63010	10.86	9.07	12.89
M3J	ON (Toronto)	50	24270	10.84	8.05	14.30
J7Y	QC	40	19470	10.81	7.72	14.72
R3M	MB	45	22090	10.72	7.82	14.35
M6J	ON (Toronto)	60	29530	10.69	8.16	13.77
L8G	ON (central)	45	22200	10.67	7.78	14.28
M1S	ON (Toronto)	70	34650	10.63	8.29	13.43
M3H	ON (Toronto)	65	32210	10.62	8.20	13.54
S4T	SK	65	32350	10.58	8.16	13.48
M1W	ON (Toronto)	100	49910	10.55	8.58	12.83
G0W	QC	80	40030	10.52	8.34	13.09
R2K	MB	65	32530	10.52	8.12	13.40
T5R	AB	50	25050	10.51	7.80	13.85

H1E	QC	85	42650	10.49	8.38	12.97
L0K	ON (central)	65	32700	10.46	8.07	13.33
M6H	ON (Toronto)	90	45300	10.46	8.41	12.85
M6G	ON (Toronto)	65	32970	10.38	8.01	13.23
R2W	MB	55	27960	10.35	7.80	13.48
J9X	QC	55	28000	10.34	7.79	13.46
M4J	ON (Toronto)	70	35710	10.32	8.04	13.04
M6M	ON (Toronto)	80	40870	10.30	8.17	12.82
M1T	ON (Toronto)	65	33240	10.29	7.94	13.12
J0Z	QC	75	38540	10.24	8.06	12.84
V1T	BC	60	31040	10.17	7.76	13.10
M6K	ON (Toronto)	65	33900	10.09	7.79	12.86
R0C	MB	95	49560	10.09	8.16	12.33
M3N	ON (Toronto)	85	44820	9.98	7.97	12.34
T0B	AB	110	60550	9.56	7.86	11.52
J0K	QC	240	132200	9.55	8.38	10.84
G0S	QC	120	68000	9.29	7.70	11.11

B: Zero Incidence

FSA	Province	Frequency	Population	Incidence per 100,000 individuals per year	Lower CI (95%)	Upper CI (95%)
V8B	BC	0	5950	0	0	3.24
J9B	QC	0	6560	0	0	2.94
J5B	QC	0	7370	0	0	2.62
G3B	QC	0	7430	0	0	2.60
G3C	QC	0	7660	0	0	2.52
G3H	QC	0	7810	0	0	2.47
T3M	AB	0	7950	0	0	2.43
V9Z	BC	0	15610	0	0	1.24
A0A	NF	0	53640	0	0	0.36

Table S3. List of Populous Forward Sortation Areas (FSA) in Canada With High Incidence of gastric adenocarcinoma from 1992 to 2010, sorted by their association with either urban or rural areas of the country. All population numbers are rounded to the nearest ten. ASIR highlighted in yellow represent the eleven FSAs had an ASIRs ≥ 3-fold that of the national average. (NF: Newfoundland and Labrador, NS: Nova Scotia, NB: New Brunswick, QC: Quebec, ON: Ontario, MB: Manitoba, SK: Saskatchewan, AB: Alberta, BC: British Columbia).

	FSA	Province	Frequency	Population	ASIR per 100,000 Individuals per Year	Lower 95% CI	Upper 95% CI
RURAL FSA	A0A	NF	160	53640	15.70	13.36	18.33
	A0B		95	23610	21.18	17.13	25.89
	A0C		35	15380	11.98	8.34	16.66
	A0E		105	29000	19.06	15.59	23.07
	A0G		140	39840	18.50	15.56	21.83
	A0H		70	20470	18.00	14.03	22.74
	A0K		100	35830	14.69	11.95	17.87
	A0L		30	7670	20.59	13.89	29.39
	A0M		40	8850	23.79	16.99	32.39
	A0N		55	14860	19.48	14.67	25.36
	B0E	NS	100	30100	17.49	14.23	21.27
	B0H		30	11630	13.58	9.16	19.38
	B0M		45	18570	12.75	9.30	17.07
	G0B	QC	30	13320	11.85	8.00	16.92
	G0C		155	52350	15.58	13.23	18.24
	G0E		45	11790	20.09	14.65	26.88
	G0H		30	11610	13.60	9.17	19.42
	G0J		95	36050	13.87	11.22	16.96
	G0L		130	63010	10.86	9.07	12.89
	G0M		80	36030	11.69	9.27	14.54
	G0N		45	21630	10.95	7.99	14.65
	G0R		190	83590	11.96	10.32	13.79
	G0S		120	68000	9.29	7.70	11.11
	G0T		60	16790	18.81	14.35	24.21
	G0W		80	40030	10.52	8.34	13.09
	G0Y		25	9450	13.92	9.01	20.56
	J0G		40	19270	10.93	7.80	14.88
	J0K		240	132200	9.55	8.38	10.84
	J0V		60	25860	12.21	9.32	15.72
	J0Z		75	38540	10.24	8.06	12.84
	L0K	ON	65	32700	10.46	8.07	13.33
	P0A		40	15710	13.40	9.57	18.25
	R0C	MB	95	49560	10.09	8.16	12.33
	S0A	SK	130	56930	12.02	10.04	14.27
	S0G		130	62640	10.92	9.13	12.97
	T0B	AB	110	60550	9.56	7.86	11.52
	V0H	BC	110	50980	11.36	9.33	13.69
	V0S		25	9710	13.55	8.77	20.00
	V0X		50	19960	13.18	9.78	17.38
	**Mean Rural**		**3150**	**1287160**	**12.88**	**12.43**	**13.34**
URB	A1B	NF	50	17450	15.08	11.19	19.88
	A1C		35	14280	12.90	8.98	17.94

A1E		80	29400	14.32	11.36	17.82
A1V		25	10260	12.82	8.30	18.93
A1Y		15	5320	14.84	8.30	24.48
A2N		25	9110	14.44	9.34	21.32
B1A	NS	50	17680	14.88	11.05	19.62
B1S		25	8550	15.39	9.96	22.72
B2A		30	9070	17.41	11.74	24.85
B5A		35	13400	13.75	9.57	19.12
E1C	NB	65	29160	11.73	9.05	14.95
E1V		30	13220	11.94	8.06	17.05
E1X		30	12620	12.51	8.44	17.86
E2A		45	18940	12.50	9.12	16.73
E2L		30	11050	14.29	9.64	20.40
E2M		40	19130	11.01	7.86	14.99
E3N		35	14170	13.00	9.05	18.08
G1E	QC	60	25180	12.54	9.57	16.14
G1K		40	18270	11.52	8.23	15.69
G1L		50	22920	11.48	8.52	15.14
G1V		40	18170	11.59	8.28	15.78
G5C		30	12740	12.39	8.36	17.69
G5H		20	7260	14.50	8.85	22.39
G5L		70	31210	11.80	9.20	14.91
G6B		25	8280	15.89	10.28	23.46
G6G		55	20420	14.18	10.68	18.45
G6V		80	35200	11.96	9.48	14.89
G7B		40	18620	11.31	8.08	15.40
G7H		65	30260	11.31	8.72	14.41
G7X		70	27310	13.49	10.52	17.04
G8L		35	13190	13.97	9.73	19.42
H1E		85	42650	10.49	8.38	12.97
H1G		110	49110	11.79	9.69	14.21
H1H		75	34700	11.38	8.95	14.26
H1J		25	9900	13.29	8.60	19.62
H1L		70	32650	11.28	8.80	14.26
H1M		60	27510	11.48	8.76	14.78
H1P		45	17420	13.60	9.92	18.19
H1R		70	29060	12.68	9.88	16.02
H1S		50	22340	11.78	8.74	15.53
H1T		85	29600	15.11	12.07	18.69
H1X		60	28820	10.96	8.36	14.10
H1Z		80	34950	12.05	9.55	14.99
H2A		45	18510	12.80	9.33	17.12
H2B		40	17150	12.28	8.77	16.72
H2E		55	24670	11.73	8.84	15.27
H3N		70	30160	12.22	9.52	15.43
H3V		20	6550	16.07	9.81	24.82
H4G		75	28700	13.75	10.82	17.24
H4H		50	19480	13.51	10.03	17.81

H4V		55	20620	14.04	10.57	18.27
H4W		90	23300	20.33	16.35	24.99
H7G		45	20140	11.76	8.58	15.74
H7V		60	20490	15.41	11.76	19.84
H8N		55	26490	10.93	8.23	14.22
H8P		55	22410	12.92	9.73	16.81
J1G		40	19250	10.94	7.81	14.89
J1T		25	7860	16.74	10.83	24.71
J1X		55	26440	10.95	8.25	14.25
J3R		30	13310	11.86	8.00	16.94
J4K		60	25480	12.39	9.46	15.95
J6E		85	38200	11.71	9.35	14.48
J6S		50	22830	11.53	8.55	15.20
J7Y		40	19470	10.81	7.72	14.72
J8H		30	13050	12.10	8.16	17.27
J9E		20	8100	13.00	7.93	20.07
J9X		55	28000	10.34	7.79	13.46
J9Z		20	8090	13.01	7.94	20.10
L2E		45	21410	11.06	8.07	14.80
L2T		25	10780	12.21	7.90	18.02
L3K		40	18660	11.28	8.06	15.36
L8G		45	22200	10.67	7.78	14.28
L8K		80	32510	12.95	10.27	16.12
L8R		30	10650	14.83	10.00	21.17
L8T		45	19270	12.29	8.96	16.45
M1R		65	28730	11.91	9.19	15.18
M1S		70	34650	10.63	8.29	13.43
M1T		65	33240	10.29	7.94	13.12
M1W		100	49910	10.55	8.58	12.83
M2M		80	29990	14.04	11.13	17.47
M2R		110	38430	15.06	12.38	18.16
M3H		65	32210	10.62	8.20	13.54
M3J		50	24270	10.84	8.05	14.30
M3M	ON	60	23730	13.31	10.15	17.13
M3N		85	44820	9.98	7.97	12.34
M4A		30	13670	11.55	7.79	16.49
M4J		70	35710	10.32	8.04	13.04
M6A		40	18530	11.36	8.12	15.47
M6C		55	24730	11.71	8.82	15.24
M6E		85	39790	11.24	8.98	13.90
M6G		65	32970	10.38	8.01	13.23
M6H		90	45300	10.46	8.41	12.85
M6J		60	29530	10.69	8.16	13.77
M6K		65	33900	10.09	7.79	12.86
M6L		55	20950	13.82	10.41	17.99
M6M		80	40870	10.30	8.17	12.82
M6N		85	41110	10.88	8.69	13.46
M8Z		35	13670	13.48	9.38	18.74

M9B		60	28220	11.19	8.54	14.40
M9C		85	35630	12.56	10.03	15.53
M9M		45	19980	11.85	8.65	15.86
M9P		45	20350	11.64	8.49	15.57
P3E		60	26580	11.88	9.07	15.29
P6B		50	23620	11.14	8.27	14.69
P7B		70	30130	12.23	9.53	15.45
R2H		45	15480	15.30	11.16	20.47
R2K		65	32530	10.52	8.12	13.40
R2V		70	30570	12.05	9.39	15.23
R2W	MB	55	27960	10.35	7.80	13.48
R2X		45	16360	14.48	10.56	19.37
R3M		45	22090	10.72	7.82	14.35
R7N		25	10090	13.04	8.44	19.25
S4H		25	10980	11.98	7.75	17.69
S4P	SK	30	12480	12.65	8.53	18.06
S4T		65	32350	10.58	8.16	13.48
T1A		55	26160	11.07	8.34	14.40
T2G		25	10900	12.07	7.81	17.82
T5B		35	15890	11.59	8.07	16.12
T5E		65	30550	11.20	8.64	14.27
T5L	AB	45	16390	14.45	10.54	19.34
T5R		50	25050	10.51	7.80	13.85
T6C		40	19230	10.95	7.82	14.91
T9C		20	7010	15.02	9.17	23.19
V1R		30	9950	15.87	10.70	22.65
V1T		60	31040	10.17	7.76	13.10
V1Y		80	31970	13.17	10.44	16.39
V2A		75	35040	11.27	8.86	14.12
V5H		70	32850	11.22	8.74	14.17
V5M	BC	45	21790	10.87	7.93	14.54
V6A		55	16530	17.51	13.19	22.79
V8A		45	17930	13.21	9.63	17.68
V8S		40	16630	12.66	9.04	17.24
V8V		50	23770	11.07	8.22	14.60
V9K		30	13530	11.67	7.87	16.66
**Mean Urban**		**7040**	**3073100**	**12.06**	**11.78**	**12.34**
